# High-Performance Hydrogel Adsorbent Based on Cellulose, Hemicellulose, and Lignin for Copper(II) Ion Removal

**DOI:** 10.3390/polym13183063

**Published:** 2021-09-10

**Authors:** Shuang Shan, Xiao-Feng Sun, Yangyang Xie, Wenbo Li, Tiezheng Ji

**Affiliations:** 1 Shenzhen Research Institute, Northwestern Polytechnical University, Shenzhen 518057, China; shanshuang00@mail.nwpu.edu.cn; 2 School of Chemistry and Chemical Engineering, Northwestern Polytechnical University, Xi’an 710129, China; yangyang636@mail.nwpu.edu.cn (Y.X.); tzji@nwpu.edu.cn (T.J.); 3 Queen Mary University of London Engineering School, Northwestern Polytechnical University, Xi’an 710129, China; liwenbo666@mail.nwpu.edu.cn

**Keywords:** cellulose, hemicellulose, lignin, hydrogel, adsorption

## Abstract

Cellulose, hemicellulose, and lignin are three kinds of biopolymer in lignocellulosic biomass, and the utilization of the three biopolymers to synthesize hydrogel adsorbent could protect the environment and enhance the economic value of the biomass. A novel hydrogel adsorbent was prepared using cellulose, lignin, and hemicellulose of wheat straw by a one-pot method, and the adsorbent showed excellent adsorption performance for copper(II) ions. Scanning electron microscopy and Fourier transform infrared spectroscopy analysis showed that the prepared straw-biopolymer-based hydrogel had porous structure, and cellulose fibrils had crosslinked with lignin and hemicellulose by poly(acrylic acid) chains. The effects of contact time, initial concentration, and temperature on the copper(II) ion removal using the prepared hydrogels were investigated, and the obtained results indicated that the adsorption kinetics conformed to the pseudo-second-order and Elovich equation models and the adsorption isotherm was in accord with the Freundlich model. The adsorption thermodynamics study indicated that the adsorption process was spontaneous and accompanied by heat. X-ray photoelectron spectroscopy analysis revealed that the adsorption behavior resulted from ion exchange. The prepared hydrogel based on cellulose, hemicellulose, and lignin could be used for water treatment and soil remediation because of its high performances of excellent heavy metal ion removal and water retention.

## 1. Introduction

In 2013, haze events triggered both public anxiety and official concerns in China, and straw burning is one of the important reasons for haze events [1]. Annual yield of agricultural straw is about 600 Tg, and 23% of crop straw was burnt before the restriction of straw burning in China [2], but most straw is returned into the soil now as fertilizer. However, the degradation of straw in soil is slow, and plant diseases and insect pests are also brought into the soil environment, and farms prefer burning straw rather than returning it into soil. The ecological environment project of China requires the 100% utilization of straw with reduced carbon release in many provinces, such as Shaanxi province, and it becomes a serious problem to enhance the economic value of straw by simple methods. Incorrect straw treatments had caused a lot of waste of resources and brought about environmental pollution [3]. Therefore, highly available and environmental approaches to utilize straw should be explored for the targets of “carbon peak” (2030) and “carbon neutrality” (2060) of China.

Wheat straw comprises hemicellulose (38.0–38.8%), cellulose (36.5–38.6%), and lignin (12.3–17.6%) [4]. Lignin molecule contains hydroxyl and carboxyl groups, which have the chelating ability, and it is beneficial for adsorbing metal ions [5]. Hemicellulose also contains a lot of hydroxyl groups and some carboxyl groups, and it could crosslink with other polymers to form a hydrogel [6,7]. The addition of lignin in the hemicellulose hydrogel was found to improve pore structure, because lignin molecule has a lot of hydroxyl groups [8], and the impact of lignosulfonates on the properties of hemicellulose hydrogel has been studied [9,10] and the mechanical, thermal, and chemical properties, as well as adsorption capacity, were dependent on lignosulfonates dosage. The presence of cellulose in composite hydrogel can significantly improve the mechanical property of the hydrogel because of strong hydrogen bonds among cellulose polymers [11,12,13]. Cellulose hydrogels have been extensively studied in wastewater treatment [14], tissue engineering [15], biomedical engineering [16], and so on. Some hydrogels have been prepared using a single biopolymer of wheat straw, such as hemicellulose [6] and lignin [17]. To improve the performance of hydrogels, using all biopolymers of straw to design and synthesize hydrogel could be a method, and all straw-biopolymer-based hydrogels could be a good adsorbent in the field of wastewater treatment because of its biocompatibility, easy degradability, and environmental friendliness. Moreover, the utilization of all biopolymers of straw to synthesize high-performance adsorbent can avoid environmental pollution and reduce the process cost.

Heavy metal pollution, which causes numerous diseases and disorders, has gradually arisen as a severe environmental problem [18,19]. Copper(II) ion pollution is a common problem in wastewater and polluted soil [19,20], and improper disposal of Cu(II) ions has caused serious environmental pollution, which limits the utilization of surface water and groundwater [21]. The traditional methods for metal ion removal involve adsorption [22], chemical precipitation [23], reverse osmosis [24], ion exchange [25], and so on, among which adsorption was thought to be an effective method to remove heavy metal ions in wastewater [21,26]. Adsorbent materials play a key role in the adsorption process, and using natural materials in water treatment has drawn great attention and a growing number of adsorbents derived from low-cost natural products have been reported [27].

In this work, a novel hydrogel adsorbent was synthesized using cellulose, hemicellulose, and lignin of wheat straw by a one-pot method, and the prepared hydrogel was used for removing copper(II) ions from aqueous solution. The morphology and chemical structure of the straw-biopolymer-based hydrogel adsorbent were analyzed by SEM, FT-IR, and XPS, and the swelling property was also studied. The effects of adsorption time, initial copper solution concentration, and temperature on adsorption behavior of the hydrogel adsorbent were examined. The adsorption isotherm, thermodynamics, and kinetics of Cu(II) ions on the straw-biopolymer-based hydrogel were also discussed.

## 2. Materials and Methods

### 2.1. Materials and Chemicals

Wheat straw was collected from Xi’an city of China and ground to pass a 1 mm size screen. Hydrogen peroxide, anhydrous sodium sulfite, and potassium persulfate were produced from the Tianjin Fuchen Chemical Reagent Co. Ltd. in China. Copper sulfate pentahydrate and ditiocarb sodium were obtained from the Tianjin Kermel Chemical Reagent Company in China. Acrylic acid and *N,N*-methylene bisacrylamide (BIS) were provided by the Tianjin Dongli District Tianda Chemical Reagent Company (Tianjin, China). All reagents used were of analytical grade.

### 2.2. Synthesis and Characterizations

#### 2.2.1. Preparation of Straw-Biopolymer-Based Hydrogel by a One-Pot Method

Wheat straw powder was first treated using hydrogen peroxide (3%, *m*/*v*) and sodium hydroxide (2%, *m*/*v*) at 65 °C for 24 h, and the obtained aqueous solution was sonicated at 650 watts for 30 min by a sonicator (JY98-IIIN, Scientz, Ningbo, China). Finally, the aqueous solution was neutralized to pH 8 for further use. The obtained aqueous solution contained cellulose (17.96 g/L), hemicellulose (10.51 g/L), and lignin (2.44 g/L), which was determined by a general straw separation method [28]. The molecular weights of the cellulose, hemicellulose, and lignin biopolymers in the solution were 385 kDa, 35 kDa, and 3730 Da, respectively, and the hemicellulose polymer contained 72.6% xylose and other sugars.

Using a free-radical polymerization method can prepare straw-biopolymer-based hydrogel. The aqueous solution (15 mL) containing all biopolymers was heated to 60 °C and stirred. Redox initiators potassium persulfate and anhydrous sodium sulfite (0.06 g) were added to the aqueous solution and stirred for 10 min, followed by adding some acrylic acid and *N*,*N′*-methylenebisacrylamide (0.025 g) to the solution and allowing it to react for 3 h. The formed gel was kept at room temperature for one night until composite hydrogel was fully formed, and then the hydrogel was immersed in water at 25 °C for 72 h. To remove the residual monomers, the water was replaced periodically. The obtained hydrogel adsorbent was oven-dried at 60 °C for 24 h.

#### 2.2.2. Characterization of Straw-Biopolymer-Based Hydrogel

An FTIR spectrometer (Nicolet 510, Durham, NH, USA) was used to analyze the chemical structure of the obtained hydrogel. An SEM instrument (VEGA 3 LMH, TESCAN, Brno, Czech) was used to observe the morphology of the hydrogel. XPS (Axis Ultra, Kratos Analytical Ltd., Stretford, UK) with an Al Kα X-ray source (1486.71 eV of photons) was also used to analyze the composite hydrogel with adsorbed copper(II) ions. All binding energies used the neutral C 1s peak at 284.6 eV as a reference to compensate for the surface charging effects.

#### 2.2.3. Swelling Test

A total of 50–100 mg of the hydrogel samples were swelled in 100 mL of ultra-pure water at 25 °C. At regular time intervals, the hydrogel samples were gathered, followed by weighing after removing redundant water on the surface using a filter paper. The swelling ratio of all the samples was tested 3 times. Equation (1) was used to calculate the swelling ratio (g/g) at time *t*:

(1) $$S_{t}=(W_{t}-W_{d})/W_{d}$$

where *W_d_* (g) and *W_t_* (g) are the weight of the dry hydrogel sample and the weight of the swollen hydrogel sample at time *t* (min), respectively.

### 2.3. Adsorption Test

#### 2.3.1. Determination of Copper(II) Ion Concentration

Copper(II) ion concentration in aqueous solution was determined according to the method stated in a previous study [29], following the equation: *A* = 0.00424*ρ* − 0.01643 (R^2^ = 0.999, *n* = 7), among which *ρ* is the concentration of copper sulfate solution and *A* is the absorbance.

#### 2.3.2. Batch Adsorption Experiment

A certain mass of hydrogel sample was added to 100 mL copper sulfate solution with a certain concentration at pH 5.5 and immersed for a period. The pH and ionic strength of each solution used for adsorption was regulated by 0.1 M HCl or NaCl solution, respectively, followed by analyzing the copper sulfate concentration of the solution. Then, Equation (2) was used to calculate the adsorption amount of copper (II) ions, *q* (mmol/g):

(2) $$q=\frac{(C_{o}-C_{t})V}{m\times 249.5}$$

where *C_o_* (g/L) is the initial concentration of the copper sulfate solution, and *C_t_* (g/L) is the concentration of the copper sulfate solution at time t; *m* (g) is the weight of the dry hydrogel, and *V* (L) is the volume of copper sulfate solution, and 249.5 is the molecular mass of copper sulfate pentahydrate.

#### 2.3.3. Desorption Experiment

To test the desorption and reusability of the obtained straw-biopolymer-based hydrogel, the used hydrogel samples with adsorbed copper(II) ions were stood in 100 mL NaCl solution, followed by stirring for 24 h at a certain temperature. Equation (3) was used to calculate desorption rate (*Ds*):

(3) $$D_{s}=\frac{C_{d}^{}}{C_{0}-C_{e}}$$

where *C_d_* is the Cu(II) ion concentration in elution (mmol/L), and *C*_0_ and *C_e_* are the initial concentration and the equilibrium concentration of copper ions (mmol/L), respectively.

The regenerated hydrogel sample was reused for six cycles of adsorption–desorption tests. Equation (4) was used to express the regeneration efficiency (*E_R_*,%), which compared the adsorption property of fresh and regenerated samples:

(4) $$E_{R}=\frac{q_{R}}{q_{F}}\times 100\%$$

where *q_R_* and *q_F_* are the amount of adsorbed copper(II) ions (mg/g) of regenerated and fresh sample, respectively.

## 3. Results

### 3.1. Preparation of Straw-Biopolymer-Based Hydrogel by a One-Pot Method

**Figure 1:**
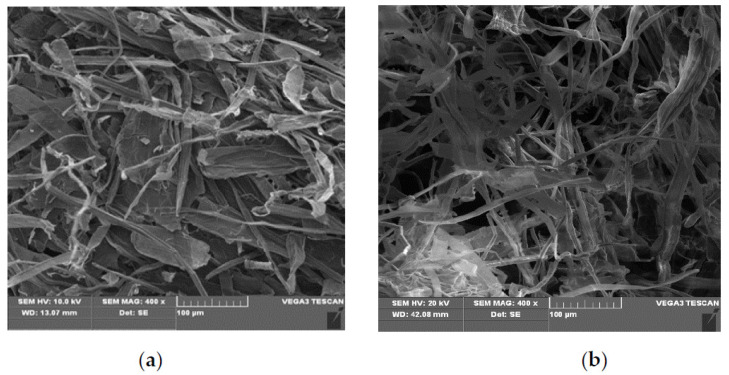
SEM images of cellulose fibers before (**a**) and after (**b**) ultrasonic irradiation.

Straw-biopolymer-based hydrogel was prepared directly using the aqueous solution containing cellulose (17.96 g/L), hemicellulose (10.51 g/L), and lignin (2.44 g/L) by a one-pot method. In order to enhance the reactivity of cellulosic microfiber, 650 watts of ultrasonic irradiation was applied to the aqueous solution, and SEM images indicated that cellulose microfibrils became smaller and appeared less bundled (Figure 1).

**Figure 2:**
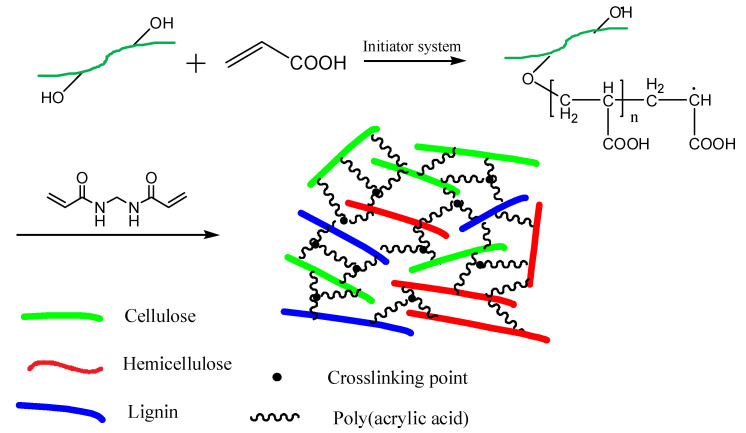
The preparation diagram of straw-biopolymer-based hydrogel.

**Figure 3:**
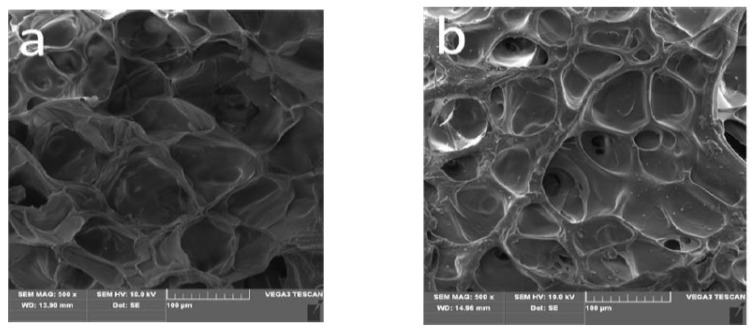
SEM images of the prepared hydrogel: (**a**) before adsorption; (**b**) after adsorption.

The proposed mechanism for synthesizing straw-biopolymer-based hydrogel is shown in Figure 2. Firstly, the hydrogen atoms derived from the hydroxyl groups of lignin, hemicellulose, and cellulose were captured by the redox initiator system (NH_4_)_2_S_2_O_8_–Na_2_SO_3_, consequently generating free radicals as active sites, and acrylic acid was grafted to the chains of lignin, hemicellulose, and cellulose, and this has been proved in previous studies [6,17,30]. After the *N*,*N′*-methylenebisacrylamide was added, a three-dimensional network structure hydrogel was formed because the cross-linker could react with two active sites. Figure 3 shows the SEM images of the prepared straw-biopolymer-based hydrogel, and it can be observed that the prepared hydrogel has a smooth surface and porous structure, and it did not show unreacted cellulose fibrils, which had crosslinked with other polymers.

### 3.2. FT-IR Spectrum of the Prepared Biopolymer Hydrogel

**Figure 4:**
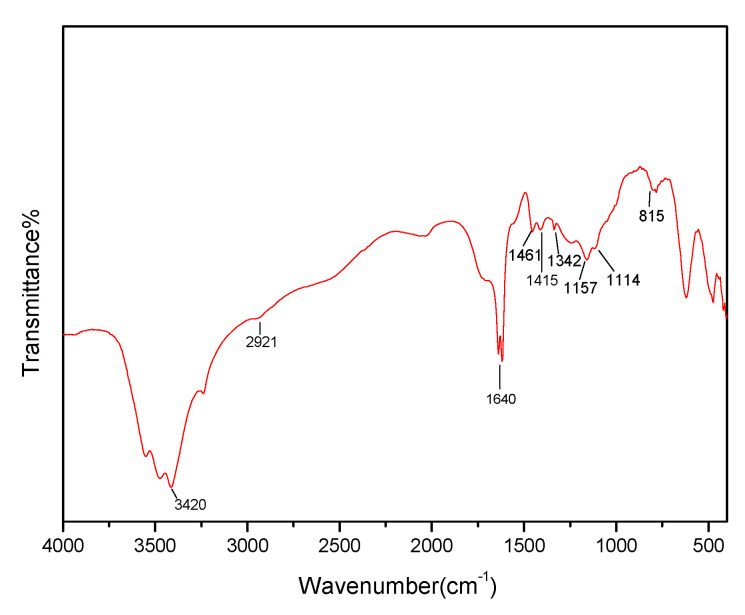
FT-IR spectrum of the prepared straw-biopolymer-based hydrogel.

FT-IR spectroscopy is used to identify the presence of certain functional groups in molecules [31]. Figure 4 shows the FT-IR spectrum of the obtained straw-biopolymer-based hydrogel. The absorption peaks between 3200 and 3650 cm^−1^ were originated from the O–H stretching of cellulose, hemicellulose, and lignin. The peak observed at 2921 cm^−1^ corresponded to the stretching band of C–H in poly(acrylic acid) and straw biopolymers. The peaks at 1461 and 1342 cm^−1^ in FT-IR spectrum corresponded to the characteristic absorptions of methyl groups in lignin, and the characteristic absorption band at 1157 cm^−1^ corresponded to the hemicellulose and cellulose, which was ascribed to the polysaccharide unit ether chain (C–O–C). The FT-IR spectrum also displays the frequency vibrations of the C1 group and pyranose ring, which corresponded to the characteristic absorption of the β-glycosidic bond between sugar units. The characteristic absorption bands at 1415 and 1114 cm^−1^ corresponded to the stretching band of the aromatic nucleus and ether linkage in lignin, respectively. The spectrum of the obtained hydrogel had an obvious absorption peak at 1640 cm^−1^ that originated from the C=O stretching vibration in poly(acrylic acid) [32], which suggested that straw-biopolymer-based hydrogel was successfully prepared from cellulose, hemicellulose, and lignin that crosslinked by poly(acrylic acid) chains.

### 3.3. Analysis of Swelling Kinetics

**Figure 5:**
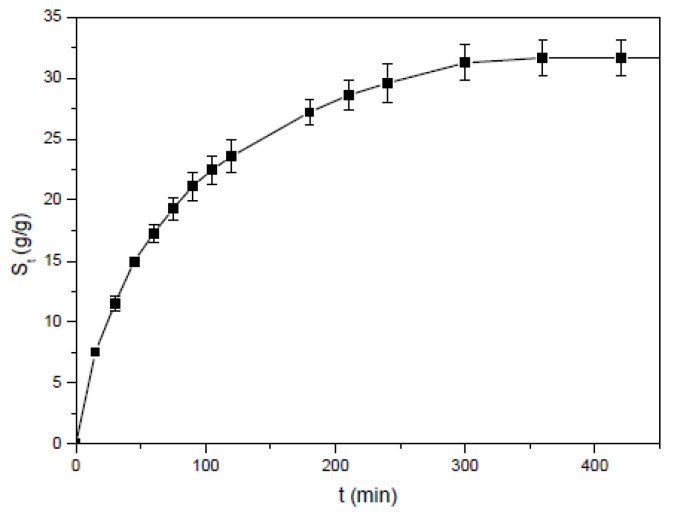
Effect of time on the swelling ratio of the hydrogel at 293 K.

The swelling property of the straw-biopolymer-based hydrogel prepared from cellulose, hemicellulose, and lignin may be different from normal hydrogel, and the swelling property will influence its application, since the swelling ratio could affect the adsorption capacity, and previous study indicated that the high swelling ratio of the hydrogels was beneficial to the diffusion of metal ions into the hydrogel and improved the adsorption performance [5,10,17]. Figure 5 shows the influence of time on the swelling ratio of straw-biopolymer-based hydrogel. Because the hydrogel was composed mainly of cellulose, hemicellulose, and lignin, the swelling ratio was relatively high at 293 K. The obtained experimental data were analyzed by using two empirical equations, including the Fickian diffusion equation and the Schott second-order dynamic equation for the explanation of the swelling mechanism of the obtained hydrogel.

The Fickian diffusion equation is often used to study the initial swelling process and diffusion mechanism of water molecules in a hydrogel [33]. It can be written as follows:

(5) $$F=\frac{S_{t}}{S_{\infty }}={kt}^{n}$$

Equation (5) can be reformed into Equation (6):

(6) $$\mathrm{In}\frac{S_{t}}{S_{\infty }}=\mathrm{In}k+n\mathrm{In}t$$

where *F* is the fractional uptake; *S_t_* and *S_∞_* are the absolute cumulative water penetrated into the hydrogel at time *t* and infinite time, respectively; *n* is the diffusion index characterizing the mechanism of water migration in the matrix; and *k* is a constant characterizing the structural and geometric properties of the matrix.

**Figure 6:**
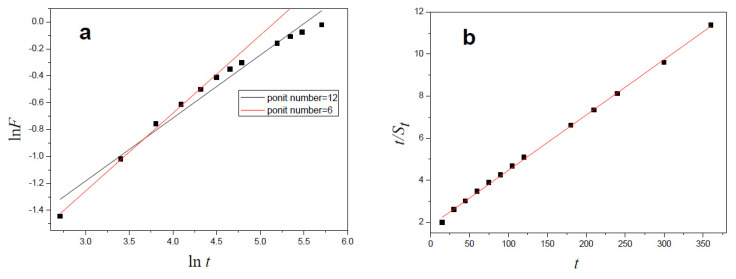
Swelling kinetic curves fitted using Fickian diffusion equation (**a**) and Schott second-order dynamic equation (**b**).

The *R*^2^ values of the two Fickian fitting curves (Figure 6a) were 0.9964 (*n* = 6) and 0.9722 (*n* = 12), respectively. Because the Fickian diffusion equation is appropriate for the initial swelling process, the *R*^2^ value of the curve (*n* = 6) fitted using initial swelling data was higher than that of the fitting curve (*n* = 12). The value of *n* that is the slope of fitting curve (*n* = 6) was 0.5787, which is between 0.5 and 1, indicating that the swelling behavior of the straw-biopolymer-based hydrogel was primarily due to the collaborative diffusion and water spread of the hydrogel, and it also indicated that the swelling kinetics of the hydrogel did not apply to Fickian diffusion. This could be explained by the first-order dynamic equation of Fickian diffusion, which presumes that the film thickness and diffusivity remain unchanged during the swelling process. The values of *R*^2^ obtained from two fitting curves (*n* = 6; *n* = 12) also proved this point.

To clearly investigate the swelling kinetics of straw-biopolymer-based hydrogel, the swelling data were analyzed by the Schott second-order kinetic equation [34]. It can be written as follows:

(7) $$\frac{t}{S_{t}}=A+Bt$$

where *B* = 1/*S_eq_*, *S_eq_* is the theoretical equilibrium swelling rate of a hydrogel; *A* = 1/*k*, *k* is the initial swelling ratio of a hydrogel.

The *R*^2^ value of the Schott fitting curve (Figure 6b) was 0.9988, and the result suggested that the swelling kinetics of the prepared hydrogel were in accordance with the Schott second-order dynamic equation.

### 3.4. Effect of Contact Time on Adsorption Behavior of the Hydrogel

**Figure 7:**
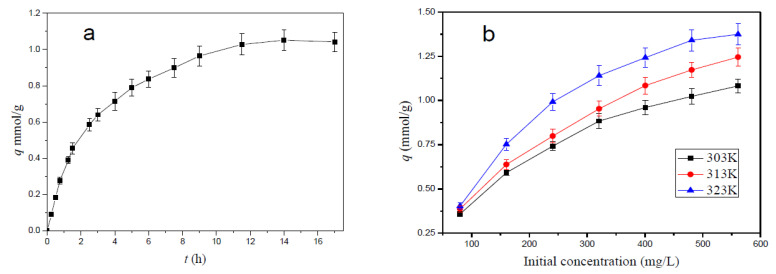
Effects of contact time (**a**) and initial concentration (**b**) on copper ion adsorption onto the hydrogel.

The effect of contact time on copper ion adsorption onto straw-biopolymer-based hydrogel is shown in Figure 7a. The result suggested that the adsorption amount of Cu(II) ions rose from 0.09 to 1.05 mmol/g with an extension of contact time from 0.25 h to 14 h (adsorbent dosage: 0.20 g/L; copper ion solution: 400 mg/L), and the adsorption amount increased rapidly within the first 4 h, and then the adsorption slowed down and remained stable. This could be ascribed to an interaction happening between the adsorption sites on the hydrogel surface and the copper ions during the initial several hours, and continuous adsorbing of the copper ions only occurred in its internal adsorption sites, causing a slow growth rate of adsorption amount.

To clearly investigate the adsorption process, the obtained experimental data were analyzed by using four kinetics adsorption models: the pseudo-first-order kinetic model, the pseudo-second-order kinetic model [35], the Elovich equation model [36], and the intraparticle diffusion model.

The pseudo-first-order kinetic model:

(8) $$\mathrm{lg}(q_{e}-q_{t})=\mathrm{lg}q_{e}-\frac{k_{1}}{2.303}t$$

The pseudo-second-order kinetic model:

(9) $$\frac{t}{q_{t}}=\frac{1}{k_{2}q_{e}{}^{2}}+\frac{1}{q_{e}}t$$

Intraparticle diffusion model:

(10) $$q_{t}=K_{p}t^{1/2}+C$$

Elovich equation model:

(11) $$q_{t}=\frac{1}{b}\ln(ab)+\frac{1}{b}\ln t$$

where *t* is the adsorption time (h), *q_t_* and *q_e_* are the adsorption amount of hydrogel sample at time *t* and infinite time, respectively (mmol/g), and *k*_1_, *k*_2_, *a*, *b*, and *K_p_* are the rate constants of the corresponding models.

**Figure 8:**
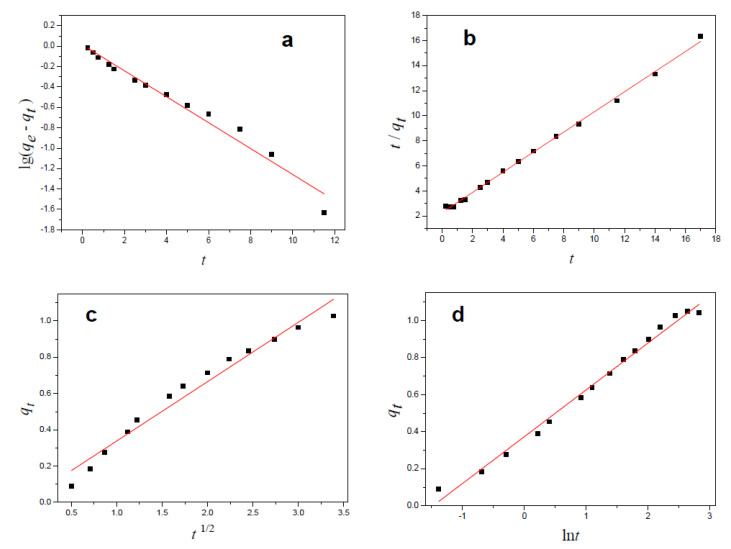
Plots of the pseudo-first-order model (**a**), pseudo-second-order model (**b**), intraparticle diffusion model (**c**), and elovich equation (**d**).

Figure 8 shows the fitted plots of the above four equations, and Table 1 lists the kinetic factors for Cu(II) ion adsorption, which were obtained by fitting experimental data to the four kinetics models. It can be observed that the pseudo-second-order and Elovich equation models (R^2^ > 0.99) were more applicable for explaining the adsorption process after comparing the correlation coefficient *R*^2^ of the four fitting curves. The result demonstrated that the adsorption process included the adsorption of copper ions on the surface of the hydrogel, the diffusion of copper ions into the active sites of the hydrogel and the final adsorption equilibrium process, and the copper(II) ion adsorption on the obtained hydrogel was due to chemisorption, and the active sites were heterogeneous, which may result from poly(acrylic acid), cellulose, hemicellulose, and lignin in the hydrogel.

### 3.5. Effect of Initial Concentration and Adsorption Isotherm

The effect of the initial concentration of copper ion solution on adsorption is shown in Figure 7b. The results indicated that the adsorption amount of copper ions on the prepared hydrogel adsorbent increased with an increase in the initial concentration (adsorbent dosage: 0.20 g/L; contact time: 48 h). This is because higher initial concentration results in more interactions between active sites and copper ions, and the phenomena is also because a higher copper ion concentration can speed up the diffusion of copper ions into the adsorbent due to a growth in the driving force of the concentration gradient.

To investigate the adsorption isotherms, the experimental data were analyzed by using three experience isothermal adsorption models, including the Freundlich isothermal adsorption model [37], the Langmuir isothermal adsorption model [38], and the Temkin isothermal adsorption model [39,40].

Freundlich equation:

(12) $$\mathrm{lg}q_{e}=\mathrm{lg}K_{F}+\frac{1}{n}\mathrm{lg}C_{e}$$

Langmuir equation:

(13) $$\frac{C_{e}}{q_{e}}=\frac{C_{e}}{q_{\max}}+\frac{1}{K_{L}q_{\max}}$$

Temkin equation:

(14) $$q_{e}=A+B\ln C_{e}$$

where *q_max_* is the theoretical maximum adsorption amount (mmol/g); *K_L_* and *K_F_* are the Langmuir constant and the Freundlich constant (mmol^1−n^·g^−1^·L^−n^), respectively; and *A* and *B* are the Temkin constants, and *n* is indicator of adsorption intensity.

By fitting experimental data to the above adsorption isotherm models, the correlation coefficients and adsorption parameters are listed in Table 2. It is clear that Freundlich isothermal model is suitable to the adsorption behavior by comparing the *R*^2^ values in Table 2, indicating that copper ion adsorption onto the obtained hydrogel tended to be multilayer adsorption [41]. *K_F_* represents the relative size of the adsorption amount, and the increase in *K_F_* value with an increase in temperature proved that the adsorption was an endothermic process. All *n* values were greater than 1, demonstrating that copper ion adsorption onto the prepared straw-biopolymer-based hydrogel was favorable, i.e., copper ions were easily adsorbed on the adsorbent. The maximum adsorption amount obtained by the Langmuir isotherm equation was 1.141 mmol/g at 303 K, and it increased to 1.421 mmol/g after increasing temperature to 323 K, and the results indicated that the higher temperature is beneficial to the adsorption. The maximum adsorption amount of copper(II) ions onto the prepared straw-biopolymer-based hydrogel was much higher than that of wheat straw, cellulose-*g*-poly(acrylic acid), and lignin-*g*-poly(acrylic acid), and the values of *q_max_* using wheat straw, cellulose-*g*-poly(acrylic acid), and lignin-*g*-poly(acrylic acid) were 0.18, 0.239, and 0.75 mmol/g, respectively [42,43,44].

### 3.6. Adsorption Thermodynamics

On the basis of the Van ‘t Hoff equation, the adsorption enthalpy change (Δ*H*) can be calculated by Equation (15):

(15) $$\ln(C_{e})=-\ln K_{0}+(\Delta H/RT)$$

where *C_e_* is the remaining Cu(II) ion concentration at adsorption equilibrium (mol/L); *K*_0_ is a constant; *T* is thermodynamic temperature; and *R* is ideal gas constant.

The adsorption enthalpy change (Δ*H*) is calculated by the slope of ln (*C_e_*) on *T* mapping. When the equilibrium adsorption quantity *q_e_* was 0.8 mmol/g, Δ*H* = 54.12 kJ/mol; when *q_e_* = 0.9 mmol/g, Δ*H* = 56.79 kJ/mol; and when *q_e_* = 1.0 mmol/g, Δ*H* = 50.83 kJ/mol.

The Gibbs Equation (16) was obtained from the Freundlich equation:

(16) $$\Delta G=-RT\int_{0}^{X}(\frac{q}{X})dX$$

where *X* is the molar concentration (mol/L); and *q* is the adsorption quantity (mmol/g).

According to the literature [45], Δ*G* is independent of *q*, and Equation (17) is deduced:

(17) ΔG=−nRT

where *n* is the Freundlich parameter. 

At 303 K, 313 K, and 323 K, the Gibbs free energy changes (Δ*G*) were −10.32 kJ/mol, −10.00 kJ/mol, and −12.82 kJ/mol, respectively.

The adsorption entropy change (Δ*S*) can be deduced by Gibbs-Helmholtz Equation (18):

(18) ΔS=(ΔH−ΔG)/T

Equations (17) and (18) were used to calculate relevant parameters, which are listed in Table 3.

The positive value of Δ*H* indicated that it was an endothermic adsorption process, and the adsorption resulted from the chemical adsorption. The negative changes of Gibbs free energy implied that the copper (II) ions tended to adsorb from the solution onto the surface of the hydrogel, indicating the spontaneity of the adsorption. The positive values of entropy change (Δ*S*) revealed the affinity of adsorbent toward copper ions in aqueous solutions. Therefore, temperature has an important influence on the adsorption process.

### 3.7. Desorption and Reusability

**Figure 9:**
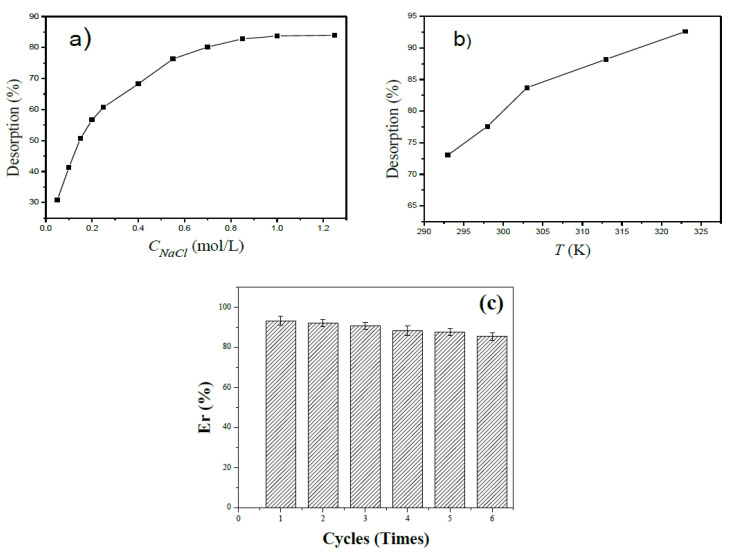
The influences of the NaCl solution concentration (**a**) and temperature (**b**) on desorption and the regeneration efficiencies of the prepared hydrogel (**c**).

The potential application value of the prepared straw-biopolymer-based hydrogel was studied based on desorption and reusability properties [46,47]. Figure 9a shows that the desorption percent of copper ions from the used hydrogel sample increased with increasing NaCl solution concentration, and the Cu(II) ions had an easy desorption from the hydrogel adsorbent by NaCl solution. This is because higher NaCl concentration led to more Na^+^ replacing the Cu^2+^ in active sites. Figure 9b presents that the desorption percent increased as the temperature rose, and the desorption behavior of copper ions favored higher temperatures. The values of desorption percent were higher than 0.83, and the highest desorption percent was 0.92; this result indicated the good performance and recyclability of the prepared straw-biopolymer-based hydrogel as an adsorbent for copper ion removal.

The reusability of the prepared straw-biopolymer-based hydrogel adsorbent was tested. Figure 9c shows that the adsorption capacity of the prepared hydrogel towards copper ions was still well-maintained, with a slight decrease after each cycle. Importantly, the regeneration efficiency (*E_R_*,%) dropped slightly from 92.1% at the first cycle to 85.3% at the sixth cycle. Such an excellent recyclability further confirmed that the straw-biopolymer-based hydrogel adsorbent may be a hopeful adsorbent to remove heavy metal ions from aqueous solution.

## 4. Discussion on Adsorption Mechanism

The pH values of the Cu(II) ion solutions before and after adsorption were approximately 5.36 and 3.44, respectively, and this could indicate that copper(II) ion adsorption onto the hydrogel adsorbent may depend primarily on ion exchange. It can be found from the SEM images of the hydrogel samples before and after adsorption (Figure 3) that the pore walls in the hydrogel sample with copper ions became thicker and the pore size became smaller. This is because of the electrostatic interaction between copper ions and COO– anions and the strong adsorption of the adsorbent.

**Figure 10:**
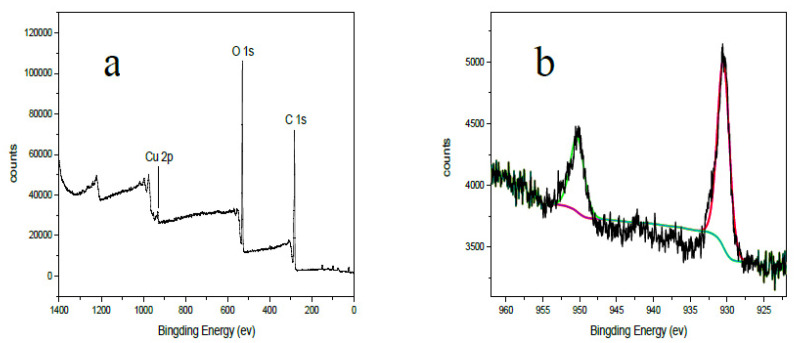
The XPS spectra of the hydrogel adsorbent after adsorption: (**a**) the wide scan spectra; (**b**) the Cu 2p spectra.

To further investigate the adsorption mechanism of the hydrogel adsorbent to Cu(II) ions, XPS was used to analyze the interactions between copper ions and the hydrogel [48]. As shown in Figure 10, a peak at the BE of 932.7 eV in the wide scan spectrum was ascribed to the Cu 2p orbital, which demonstrated that the copper ion was adsorbed on the hydrogel. There were two BE peaks at around 950.5 eV and 930.7 eV in Cu 2p spectrum, which arose from the copper ions in the hydrogel and the sulfur–oxygen bond of copper sulfate, respectively. All results showed that chemisorption was the main adsorption mechanism.

## 5. Conclusions

A straw-biopolymer-based hydrogel adsorbent was prepared successfully by a one-pot method after separating cellulose, hemicellulose, and lignin of wheat straw into aqueous solution. The swelling kinetics of the straw-biopolymer-based hydrogel was in accordance with the Schott second-order dynamic equation. The adsorption isotherm and kinetics studies implied that the Cu(II) ion adsorption onto the hydrogel conformed to the Freundlich isothermal adsorption model and the pseudo-second-order kinetic model. The adsorption thermodynamics study indicated that the adsorption process may be spontaneous and accompanied by heat. The adsorption behavior of the straw-biopolymer-based hydrogel towards Cu(II) ions resulted from ion exchange, and the prepared hydrogel adsorbent also had excellent reusability. The prepared straw-biopolymer-based hydrogel could be used for water treatment and soil remediation because of its high performances of heavy metal ion removal and water retention, and the utilization of all straw biopolymers could reduce carbon release and enhance the economic value of straw.

## Figures and Tables

**Table 1:** Kinetic parameters for copper ion adsorption on the prepared hydrogel.

Pseudo-First-Order			Pseudo-Second-Order			Intraparticle Diffusion			Elovich Equation		
*q_e_*	*k*_1_	*R* ^2^	*q_e_*	*k*_2_	*R* ^2^	*K_p_*	*C*	*R* ^2^	*a*	*b*	*R* ^2^
1.0286	0.2926	0.9711	1.2444	0.2846	0.9978	0.3269	0.0115	0.9660	1.0940	3.8939	0.9910

**Table 2:** Isotherm parameters for copper ion adsorption on the prepared hydrogel.

T	Langmuir			Freundlich			Temkin		
	K_L_	q_max_	R^2^	K_F_	n	R^2^	A	B	R^2^
303 K	0.0341	1.141	0.9863	0.2618	4.098	0.9938	0.1141	0.1583	0.9447
313 K	0.0411	1.320	0.9790	0.2743	3.842	0.9913	0.0839	0.1889	0.9256
323 K	0.0617	1.421	0.9931	0.4256	4.775	0.9949	0.3867	0.1656	0.9550

**Table 3:** Thermodynamic parameters for copper ion adsorption on the prepared hydrogel.

*q_e_* (mmol/g)	Δ*H* (kJ/mol)	Δ*S* (kJ/(mol·K))		
		303 K	313 K	323 K
0.8	54.12	0.2127	0.2048	0.2072
0.9	56.79	0.2215	0.2134	0.2155
1.0	50.83	0.2018	0.1943	0.1971

## Data Availability

Data available on request due to restrictions, e.g., privacy or ethical.
